# Impact of mHealth interventions for reproductive, maternal, newborn and child health and nutrition at scale: BBC Media Action and the *Ananya* program in Bihar, India

**DOI:** 10.7189/jogh.10.021005

**Published:** 2020-12

**Authors:** Victoria C Ward, Hina Raheel, Yingjie Weng, Kala M Mehta, Priyanka Dutt, Radharani Mitra, Padmapriya Sastry, Anna Godfrey, Melissa Shannon, Sara Chamberlain, Rajani Kaimal, Suzan L Carmichael, Jason Bentley, Safa Abdalla, Kevin T Pepper, Tanmay Mahapatra, Sridhar Srikantiah, Evan Borkum, Anu Rangarajan, Swetha Sridharan, Dana Rotz, Priya Nanda, Usha Kiran Tarigopula, Yamini Atmavilas, Debarshi Bhattacharya, Gary L Darmstadt, for the Ananya Study Group, Yamini Atmavilas, Debarshi Bhattacharya, Jason Bentley, Evan Borkum, Suzan Carmichael, Indrajit Chaudhuri, Andreea Creanga, Gary L Darmstadt, Priyanka Dutt, Laili Irani, Tanmay Mahapatra, Kala M Mehta, Radharani Mitra, Wolfgang A Munar, Priya Nanda, Kevin T Pepper, Hina Raheel, Anu Rangarajan, Niranjan Saggurti, Padmapriya Sastry, Hemant Shah, Sridhar Srikantiah, Usha Kiran Tarigopula, Victoria Ward, Yingjie Weng, Dilys Walker, Jess Wilhelm

**Affiliations:** 1 Department of Pediatrics, Stanford University School of Medicine, Stanford, California, USA; 2 Quantitative Sciences Unit, Department of Medicine, Stanford University School of Medicine, Stanford, California, USA; 3 Department of Epidemiology and Biostatistics, University of California San Francisco, San Francisco, California, USA; 4 BBC Media Action (India), New Delhi, India; 5 BBC Media Action, London, UK; 6 Center for Population Health Sciences, Stanford University School of Medicine, Palo Alto, California, USA; 7 CARE India, Patna, India; 8 Mathematica, Princeton, New Jersey, USA; 9 Bill and Melinda Gates Foundation, Delhi, India

## Abstract

**Background:**

Mobile health (mHealth) tools have potential for improving the reach and quality of health information and services through community health workers in low- and middle-income countries. This study evaluates the impact of an mHealth tool implemented at scale as part of the statewide reproductive,**maternal, newborn and child health and nutrition (RMNCHN) program in Bihar, India.

**Methods:**

Three survey-based data sets were analysed to compare the health-related knowledge, attitudes and behaviours amongst childbearing women exposed to the Mobile *Kunji* and Dr. Anita mHealth tools during their visits with frontline workers compared with those who were unexposed.

**Results:**

An evaluation by Mathematica (2014) revealed that exposure to Mobile *Kunji* and Dr. Anita recordings were associated with significantly higher odds of consuming iron-folic acid tablets (odds ratio (OR) = 2.3, 95% confidence interval (CI) = 1.8-3.1) as well as taking a set of three measures for delivery preparedness (OR = 2.8, 95% CI = 1.9-4.2) and appropriate infant complementary feeding (OR = 1.9, 95% CI = 1.0-3.5). CARE India’s Community-based Household Surveys (2012-2017) demonstrated significant improvements in early breastfeeding (OR = 1.64, 95% CI = 1.5-1.78) and exclusive breastfeeding (OR = 1.46, 95% CI = 1.33-1.62) in addition to birth preparedness practices. BBC Media Action’s Usage & Engagement Survey (2014) demonstrated a positive association between exposure to Mobile *Kunji* and Dr. Anita and exclusive breastfeeding (58% exposed vs 43% unexposed, *P* < 0.01) as well as maternal respondents’ trust in their frontline worker.

**Conclusions:**

Significant improvements in RMNCHN-related knowledge and behaviours were observed for Bihari women who were exposed to Mobile *Kunji* and Dr. Anita. This analysis is unique in its rigorous evaluation across multiple data sets of mHealth interventions implemented at scale. These results can help inform global understanding of how best to use mHealth tools, for whom, and in what contexts.

**Study registration:**

ClinicalTrials.gov number NCT02726230.

Although substantial global progress has been made in the past decade to reduce maternal and under-five child mortality, reduction in newborn deaths continues to lag [1,2]. Further, approximately 95% of maternal and child deaths occur in low- and middle-income countries (LMICs) [3], demonstrating that critical gaps and disparities remain despite the availability of numerous evidence-based reproductive, maternal, newborn and child health and nutrition (RMNCHN) interventions [4]. Impact of these interventions may be attenuated by the challenges of maintaining their quality and coverage at scale [5,6], particularly in rural and under-resourced areas with shortages of qualified health care workers [7] and limited access to antenatal care (ANC), skilled attendance at delivery, emergency obstetric care and postnatal care for neonates [8].

A growing body of research has suggested that a primary solution for improving access to quality health care is to expand frontline health worker (FLW) home visits [9-11]. The quality of these visits, however, is dependent upon supportive training, tools and supervision [12,13]. To provide this support as well as to further expand quality health care for marginalised populations in hard to reach places, perhaps no interventions have received as much enthusiasm as mobile health (mHealth) [14,15]. In the past decade, there has been a rapid expansion of communication technologies available throughout LMICs, exceeding that of many other health services [16]. This has created a new opportunity to empower FLWs and their beneficiaries with novel methods of communication, education and training. Further, the modern proliferation of mHealth tools has enabled expanded access to evidence-based interventions at scale, particularly in LMICs. This has allowed for an unparalleled opportunity to increase the reach and improve the delivery of quality health interventions and services to previously marginalised populations.

Multiple studies have assessed the impacts of mHealth interventions for maternal and newborn care in LMICs [17-23]. Free et al concluded that the use of mobile technology for clinical decision support and on-the job-training for community workers led to significant improvements in the quality of health care provided and suggested this may facilitate health systems strengthening [21]. We showed previously that use of an mHealth tool to support FLW delivery of RMNCHN health services in Bihar, India was associated with significant improvements in FLW self-efficacy as well as quality and frequency of service delivery, leading to improved health behaviours of beneficiaries [24]. Sondaal et al suggested that mHealth tools have demonstrated consistent success in increasing compliance with recommendations for ANC visits [23,25-28], and a review by Saronga et al demonstrated that mHealth interventions could be used to improve compliance with micronutrient supplementation [29]. Effects on newborn care have been more limited, although some evidence suggests that communication interventions improved postnatal care attendance [28,30], immunisation compliance [26] and exclusive breastfeeding [31,32]. Much of this evidence, however, is derived from small pilot studies which may lack methodologic rigor.

While widely considered to be promising for solving major problems of poor health and poverty, the impacts of mHealth tools have rarely been subjected to rigorous evaluation, especially for critical health behaviours at scale [22]. We hypothesised that utilisation of mHealth tools implemented at scale would improve RMNCHN-related knowledge, attitudes and behaviours amongst women in Bihar, India.

## METHODS

### Setting

In 2010, India accounted for nearly 20% of the world’s population with disproportionately high burdens of maternal and neonatal mortality, low birth weight babies and underweight children [33]. The state of Bihar is one of India’s most populous states (104 million in 2011) [34], is among its poorest with 40% of the population living below the poverty line, and had some of the worst maternal and child health indicators of any Indian state (Table S1a and S1b in the **Online Supplementary Document**) [35]. However, it also had the fastest growing economy and increasing government commitment to health, particularly maternal and child health. Further, there was a relatively high penetration of mobile phone access in Bihar, with 63% of rural men owning a mobile phone. While only 32% of rural women claimed phone ownership, 83% of women reported access to one [36].

In 2010, the Bill & Melinda Gates Foundation (BMGF) partnered with the Government of Bihar (GoB) to implement *Ananya*, a large-scale RMNCHN technical support program with the goal of achieving statewide improvement in health outcomes. Their investment relied on both supply and demand-side interventions to improve health, expanding the availability of quality health interventions while simultaneously building demand for them through improved health knowledge and behaviours [35]. New tools and interventions were designed and piloted by grantees, with subsequent provision of technical support to the GoB to facilitate statewide scale-up of those found to be successful [37,38]. A grant entitled “Shaping Demand and Practices” was awarded in 2011 to BBC Media Action, an independent charity based in the UK. While BBC Media Action had previously achieved success implementing health programs across India [36,39,40], this grant required a large-scale, multi-platform suite of interventions across the eight “focus” districts, reaching 28 million people, with plans to scale up effective interventions across all 38 districts of Bihar in 2014 [41]. While BBC Media Action‘s many mHealth tools, including multimedia campaigns and audio-based educational and communication interventions are described elsewhere [42-47], this study focuses on the impacts of the FLW job aid called Mobile *Kunji* (Table S2 in the **Online Supplementary Document**).

### Intervention: *Mobile Kunji*

BBC Media Action aimed to increase demand for health services through education and the promotion of specific health behaviours by FLWs. Mobile *Kunji* was a tool developed using principles of human-centered design as an audiovisual job aid for FLWs to improve effective and timely delivery of key health messages during their conversations with individual families [48]. The tool included a deck of 40 color-coded cards with pictures to support explanations of specific health topics. Printed on each card was a unique mobile short code used to call an Interactive Voice Response (IVR) service with a message specific to the woman’s stage of pregnancy, childbirth or childcare. When a health worker dialed the number via their mobile phone, a fictional character named “Dr. Anita” would deliver a corresponding pre-recorded audio health message. Thus, the Mobile *Kunji* deck of cards and the Dr. Anita educational messages could be used together, or separately, depending on the needs of the FLW. Other audio tools including *Kilkari,* a weekly stage-appropriate voice message delivered directly to families’ mobile phones, and Mobile Academy, a mobile-based audio training course for FLWs, are described elsewhere and are not evaluated here [42,45].

### Implementation

Similar to other interventions of the *Ananya* program [35], BBC Media Action initially piloted its tools from 2012 through 2013 in the eight focus districts. Interventions shown to be successful would then be distributed statewide to the other 30 “non-focus” districts through the transfer of ownership and implementation to the government. Statewide scale-up began in early 2014 and implementation continues through the present time.

### Data sources

In order to evaluate the impact of the Mobile *Kunji* tool, we analysed three separate data sources: Mathematica’s *Ananya* evaluation (2014) [49]; CARE India’s Community-based Household Surveys (2012-2017) [50]; and BBC Media Action’s own Usage & Engagement Study (2014). A timeline describing the periods of data collection compared with the implementation of interventions can be found in Figure S1 in the **Online Supplementary Document**.

#### Mathematica Evaluation

Mathematica implemented a household evaluation survey from January through April 2014, during the period that corresponded with the intended completion of intensive pilot testing in the eight focus districts and prior to statewide scale-up. A listing was conducted to identify eligible women for inclusion who had given birth in the previous 12 months (about 13 women per village, on average), and households were selected using a multistage sampling approach, starting with a random selection of geographic blocks in each of the 38 districts, and then a random selection of villages within each block, as described previously [35,49]. Survey data were collected by an independent contractor (Sambodhi) from maternal household respondents, who were asked whether Mobile *Kunji* and Dr. Anita had been used during FLW home visits. They were also asked questions regarding RMNCHN behaviours relevant to Mobile *Kunji *and Dr. Anita. We assessed the reach as well as the impact of Mobile *Kunji* and Dr. Anita on specific health indicators by comparing the behaviours of those exposed to the tools in the eight focus districts to those who were not exposed to them in the same districts.

#### Community-based Household Survey (CHS)

For the purposes of monitoring the reach and benefit of *Ananya* interventions, CARE India collected nine rounds of survey data from 2012 through 2017 using a methodology similar to Lot Quality Assurance Sampling (LQAS), as described previously [35,50]. We excluded round 1 in the analysis because it was used as a pilot survey. Rounds 2-5 of the CHS were conducted between September 2012 and December 2013 during the period of intensive implementation in the eight focus districts (Phase 1). Rounds 6-9 of the CHS corresponded with the statewide scale-up of interventions across all 38 districts with the support of the Bihar Technical Support Program (BTSP) (Phase 2) [35]. For rounds 2-5, eligible women were sampled in only the eight focus districts, while rounds 6-9 were conducted in all 38 districts. In each round, standardised information was collected from each selected household on all pregnancies and their outcomes, as well as RMNCHN knowledge and behaviours. In this study, we focus on the changes in health-related knowledge and behaviours for those exposed vs those unexposed to both the Mobile *Kunji* and Dr. Anita interventions.

#### Usage and Engagement (U&E) Study

In order to assess how Mobile *Kunji* and Dr. Anita were being used and whether the tools supported improvements in interactions between FLWs and beneficiary families, BBC Media Action carried out a Usage & Engagement survey. Surveys were collected from household respondents from October to December 2014 in the eight focus districts. From the catchment areas of 585 FLWs in these districts, a listing of eligible respondents was conducted and 6-7 women were randomly selected for inclusion. Women were surveyed on whether they had been exposed to the Mobile *Kunji* tool during their last two visits with a FLW, as well as on the primary topics they recalled having heard during their visits. Knowledge and attitudes versus health-related behaviours were then compared between those exposed to Mobile *Kunji* versus those unexposed to it.

### Statistical analysis

All analyses were conducted in Stata version 14 [51] and SAS 9.4 [52]. No imputation was used for missing data and all data were handled as complete case analysis.

For the Mathematica data set, we utilised a logistic regression model to assess differences in RMNCHN-related knowledge and self-reported behaviours between maternal respondents in the eight focus districts who were exposed vs those unexposed to Mobile *Kunji*. We examined the demographic characteristics of maternal respondents by whether they were exposed or unexposed and reported crude percentages without adjusting for survey design or weights. *P*-values were calculated using two-sample *t* tests for continuous variables and χ^2^ tests for categorical variables, assessed at alpha = 0.05. The demographic characteristics that were found to be statistically different between the exposed and unexposed groups were adjusted for in the model, and included maternal age, caste, number of children, socioeconomic status (SES) quartile, urban/rural residence and the household having a Below Poverty Line (BPL) card. We used survey poisson regressions for count-type indicators while survey logistic regressions were used for binary indicators. Odds ratios (ORs) with 95% confidence intervals (CIs) are reported.

For the CHS data, we reported adjusted percentages for RMNCHN-related knowledge and self-reported behaviours of maternal respondents in implementation districts (eight districts in rounds 2-5, 38 districts in rounds 6-9) who were exposed vs unexposed to Mobile *Kunji*. ORs with 95% CIs are reported with reference to the value in round 2. ORs were evaluated for round 5 compared to round 2 (difference across phase 1), round 9 compared to round 6 (difference across phase 2), and round 9 compared to round 2 (difference across the full intervention period). In an attempt to control for secular trends due to changing characteristics of the sample over time, models were adjusted for sociodemographic variables identified as potential confounders, including maternal age, religion (Hindu or not), belonging to the Scheduled Caste/Scheduled Tribe (SC/ST), number of children, gender of the focal child, household size, type of house, nuclear family, literacy and socioeconomic status (SES) quartile. Due to the large number of comparisons, we applied the False Discovery Rate (FDR) controlling procedure by Benjamini and Hochberg [53] using SAS (proc multtest) to all trend estimates together from all models, applying an upward adjustment to the *P*-values. Family-wise type I error, alpha, was controlled at 0.05. Because adjustment of the *P*-values did not affect the conclusions, we report the results with the original confidence intervals. Analyses accounted for complex survey design and sampling weights.

For the U&E study, crude percentages are reported comparing the group exposed to Mobile *Kunji* vs those unexposed to the tool. Information about survey weighting and participant demographics were not available, and therefore they were not adjusted for in this analysis. *P*-values were calculated using two-sample *t*-tests for the continuous variables and χ2 test for the categorical variables to assess the difference between the two groups.

### Ethical considerations

The *Ananya* program was registered with ClinicalTrials.gov number NCT02726230. Ethical approval for the *Ananya* intervention and subsequent analyses was received from the Institutional Review Board of the Public Health Foundation of India, and from the Health Ministry’s Screening Committee on August 18, 2011. Ethical approval for data analyses done at Stanford University was received from the Stanford Institutional Review Board on December 19, 2016, protocol ID 39719.

## RESULTS

Demographics

The mean age of respondents across all data sources was 25 years. The majority were Hindu (75%-90%) and approximately 30% belonged to SC/ST (**Table 1** and **Table 2**). For the CHS surveys, SC/ST also included women who belonged to Other Backward Class, which made up the vast majority of women in this survey (about 90%). More than half of the women had two or more children and approximately half had not received any formal education. Significant differences existed between the exposed and unexposed groups for number of children in the home across all data sets. In the CHS surveys, there were also significant differences between groups in belonging to a nuclear family, and in the second phase in maternal age, type of house and household size. All of these differences were adjusted for in the analysis. Additionally, while 36%-38% of women surveyed were literate in the first phase of surveys, 42%-44% of women were literate in the second phase.

**Table 1:** Demographic characteristics of the maternal household respondents in surveys used to evaluate the Shaping Demand and Practices BBC Media Action mHealth interventions within the *Ananya* program in Bihar, India

	Mathematica (Focal districts only)		U & E	
	**Jan-Apr, 2014**		**Oct-Dec, 2014**	
Maternal characteristics (%)*	**Exposed to Mobile *Kunji* (n = 318)**	**Unexposed to Mobile *Kunji *(n = 2774)**	**Exposed to Mobile *Kunji* (n = 2423)**	**Unexposed to Mobile *Kunji *(n = 956)**
Age in years (mean, standard deviation)	25.4 (4.3)	25.1 (4.5)	-	-
Religion:				
Hindu	78	80	88	88
Muslim	22	20	-	-
Others	-	-	12	12
Caste:				
Scheduled Caste/Tribe (SC/ST)	29	29	33	27
Other Backward Class			56	63
General Caste			10	10
Others			-	-
No formal schooling	54	51	-	-
Birth parity:				
0 or 1			37	46
2 or more			63	54
1 child	27	30		
2 children	25	28		
3 children	19	20		
4+ children	30	22		

*Percentage unless otherwise specified.

**Table 2:** Demographic characteristics of the maternal household respondents in the Community-based Household Surveys used to evaluate the Shaping Demand and Practices BBC Media Action interventions within the *Ananya* program in Bihar, India

	Rounds 2-5 (8 focus districts)			Rounds 6-9 (8 focus districts)		
**Maternal characteristics**	**Exposed (n = 1446)**	**Unexposed (n = 4463)**	***P-*value**	**Exposed (n = 1628)**	**Unexposed (n = 4688)**	***P*-value**
Mean age (years)	25.1 (4.3)	25 (4.4)	0.270	24.6 (4.1)	24.1 (4.4)	<0.001
Hindu (%)	1271 (87.9)	3967 (88.9)	0.326	1465 (90)	4134 (88.2)	0.053
SC/ST* (%)	465 (32.2)	1252 (28.1)	0.003	501 (30.8)	1202 (25.6)	<0.001
Literate (%)	538 (37.2)	1619 (36.3)	0.544	693 (42.6)	2047 (43.7)	0.459
Gender of focal child (male, %)	778 (53.8)	2323 (52.1)	0.258	861 (52.9)	2428 (51.8)	0.463
Household size (median, interquartile range, IQR)†	7.00 [5.00, 9.00]	7.00 [5.00, 10.00]	0.338	6.00 [5.00, 9.00]	7.00 [5.00, 9.00]	<0.001
By major group (%):			0.7			0.267
1	0 (0.0)	0 (0.0)		1 (0.1)	0 (0.0)	
2	2 (0.1)	5 (0.1)		1 (0.1)	5 (0.1)	
3	54 (3.7)	195 (4.4)		75 (4.6)	215 (4.6)	
4	142 (9.8)	415 (9.3)		192 (11.8)	493 (10.5)	
5+	1248 (86.3)	3848 (86.2)		1359 (83.5)	3975 (84.8)	
Number of children (median, IQR)†	2.00 (2.00, 4.00)	2.00 (1.00, 3.00)	0.019	3.00 (2.00, 4.00)	2.00([1.00, 4.00)	<0.001
By major group (%):			0.054			<0.001
1	361 (25.0)	1210 (27.1)		351 (21.6)	1266 (27.0)	
2	375 (25.9)	1241 (27.8)		426 (26.2)	1242 (26.5)	
3	321 (22.2)	940 (21.1)		380 (23.3)	945 (20.2)	
4+	389 (26.9)	1072 (24)		471 (28.9)	1235 (26.3)	
Nuclear family (%)	546 (37.8)	1567 (35.1)	0.073	723 (44.4)	1761 (37.6)	<0.001
Type of house (%):			0.503			0.011
*Kutcha*	630 (43.6)	1885 (42.2)		477 (29.3)	1265 (27.0)	
*Pucca*‡	236 (16.3)	710 (15.9)		276 (17.0)	704 (15.0)	
Semi-*pucca*	580 (40.1)	1868 (41.9)		875 (53.7)	2719 (58.0)	

IQR – interquartile range

*In CHS data, SC/ST refers to scheduled caste, scheduled tribe and other backward caste.

†Median and IQR used due to non-normal data.

‡Refers to dwellings that are designed to be solid and permanent. This term is applied to housing in South Asia built of substantial material such as stone, brick, cement, concrete or timber.

### Mathematica evaluation

#### Exposure to Mobile *Kunji*

The Mathematica midline survey in early 2014 was collected two years after the launch of Mobile *Kunji*. At that time, the reach of services was proportionally low, although it was significantly higher among women in the eight focus districts vs the 30 comparison districts (8.5% vs 1.3%, *P* < 0.001). Amongst women in the eight focus districts who reported having received a visit from a FLW during the past 6 months, and thus had an opportunity to be exposed to the tools, a significantly higher percentage of women reported that Mobile *Kunji* had been used compared with those who had received a FLW visit in the comparison districts (39.1% vs 8.6%, *P* < 0.001). Given the limited reach, the exposed group in the eight focus districts was significantly smaller (n = 318) than the unexposed group in those districts (n = 2774).

#### Impact of Mobile Kunji

The odds of receiving 90 or more iron-folic acid (IFA) tablets during pregnancy was twice as high among women in the eight focus districts who had been exposed to Mobile *Kunji* compared to those who had not been exposed (OR = 2.0, 95% CI = 1.5-2.7). Similar results were found for consumption of IFA tablets (OR = 2.3, 95% CI = 1.8-3.1) (**Table 3**). The odds of delivery preparedness practices such as saving money, identifying transport and saving important delivery-related phone numbers increased by two- to 3-fold for women who were exposed to Mobile *Kunji*. Further, women exposed to Mobile *Kunji* were nearly three times as likely to have acted on all three measures for delivery preparedness (OR = 2.8, 95% CI = 1.9-4.2). Their odds of exclusive breastfeeding were significantly higher (OR = 1.8, 95% CI = 1.3-2.7), as was their likelihood to have initiated complementary feeding by six months of age (OR = 1.9, 95% CI = 1.0-3.5) and to be implementing appropriate dietary diversity (OR = 1.3, 95% CI = 1.0-1.7). There were no significant differences in contraception or immunisation practices between the two groups. Given that not all women who were exposed to the Mobile *Kunji* tool were only in the focus districts, we additionally performed a sensitivity analysis to assess those in the exposure group compared to those in the non-exposure group regardless of whether they were in the eight focus districts or the 30 comparison districts. Similar results were found.

**Table 3:** Health behaviours of maternal household respondents exposed vs unexposed to Mobile *Kunji* in eight focus districts, Mathematica (2014) survey, Bihar, India

	Exposed to Mobile *Kunji*	Unexposed to Mobile *Kunji*	OR (95% CI)*
Sample size (n)	318	2774	
Antenatal care (%):			
Received 90+ iron-folic acid (IFA) tablets	27.8	15.9	2.0 (1.5-2.7)
Consumed 90+ IFA tablets	27.5	13.8	2.3 (1.8–3.1)
Delivery preparedness (%):			
Saved money for delivery	91.7	77.5	3.2 (1.7-5.9)
Identified transport for delivery	69.8	55.7	1.8 (1.3-2.6)
Saved important phone numbers for delivery	71.5	41.9	3.5 (2.0-6.0)
Prepared all three items above for delivery	55.4	30.9	2.8 (1.9-4.2)
Breastfeeding (%):			
Breastfed child within 1 hours of delivery	50.5	52.3	0.9 (0.7-1.3)
Breast fed child within 2 hours of delivery	69.8	67.2	1.2 (0.7-1.8)
Exclusive breast feeding for child (6-12 months)	77.6	65.1	1.8 (1.3-2.7)
Immunisation (%):			
Fully immunised, except measles	25.0	29.4	0.9 (0.6-1.1)
Contraception (%):			
Plan to use in the next 12 months	47.1	40.1	1.3 (0.8-2.2)
Current use of any modern method	24.4	19.0	1.2 (0.7-2.2)
Complementary feeding (6-11 months) (%):			
Sample size (n)	112	1197	
Began receiving any solid/semisolid food by age 6 months	79.5	67.0	1.9 (1.0-3.5)
Received recommended frequency of feeding yesterday	34.7	32.2	1.1 (0.6-2.0)
Received recommended quantity of feeding yesterday	5.8	9.3	0.7 (0.2 -2.5)
Dietary diversity index, food fed in the past 24 hours (0-6)†, mean ± sd	1.84 ± 0.16	1.42 ± 0.05	1.3 (1.0-1.7)
Food frequency index, foods fed in the past 7 days†, mean ± sd	3.08 ± 0.40	2.36 ± 0.08	1.3 (0.9 -1.8)

OR – odds ratio, CI – confidence interval, sd – standard deviation

*All models adjusted for age, SC/ST, birth parity, SES quartile, household having BPL card, urban/rural. Reference group is women who were unexposed to Mobile *Kunji* in all models. OR>1 means higher odds of the indicators for women who were exposed to Mobile *Kunji* vs women who were unexposed to Mobile *Kunji*.

†Survey-weighted Poisson regressions were used for the count indicators, risk ratios were reported.

### Community-based Household Survey (CHS)

#### Exposure to Mobile *Kunji*

**Figure 1:**
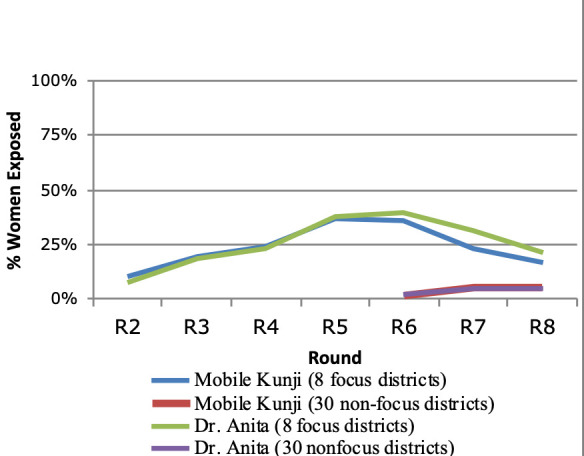
Exposure to Mobile *Kunji* and Dr. Anita in eight focus districts and thirty non-focus districts, Community-based Household Surveys, Bihar, India, 2012-2017.

Exposure to Mobile *Kunji* steadily increased in the eight focus districts during CHS survey rounds 2-5 (2012-2013) (**Figure 1**). Given that the Mobile *Kunji* job aid and the audio IVR component of Dr. Anita could be used either together or separately, the percentage of women reporting exposure to each component were compared separately and found to have similar results. Later, during rounds 6-9 in the scale-up period from 2014 to 2017, there was a decline in exposure to these tools in the eight focus districts and a small increase in exposure in the 30 non-focus districts where implementation began in round 6 (2014).

#### Impact of mHealth Tools

**Figure 2:**
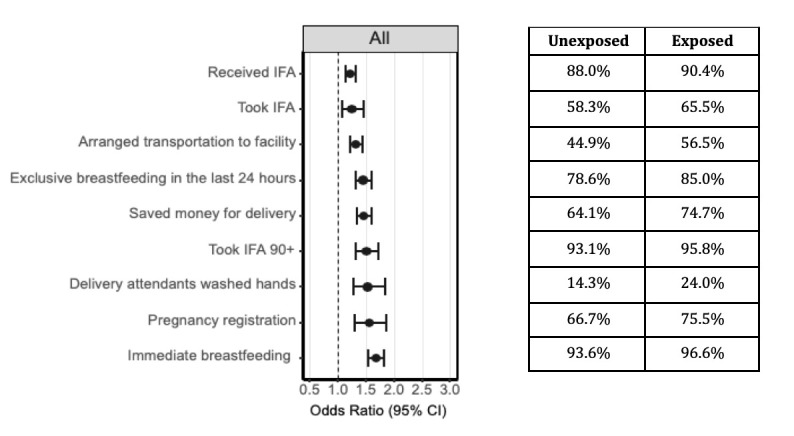
Comparison of reproductive, maternal, newborn and child health and nutrition behaviours for maternal household respondents in eight focus districts who were exposed to Mobile Kunji vs those who were unexposed across rounds 2-9 of the Community-based Household Surveys, Bihar, India, 2012-2017.

Multiple health behaviours were significantly higher in those who were exposed to Mobile *Kunji* as compared to those who were unexposed, including odds of pregnancy registration (OR = 1.64, 95% CI = 1.37-1.98), birth preparedness activities such as saving money for delivery (OR = 1.5, 95% CI = 1.37-1.64) and arranging transport to the facility (OR = 1.33, 95% CI = 1.22-1.44), receipt (OR = 1.29, 95% CI = 1.2-1.4) and consumption (OR = 1.3, 95% CI = 1.1-1.51) of IFA tablets, as well as immediate breastfeeding (OR = 1.64, 95% CI = 1.5-1.78) and exclusive breastfeeding (OR = 1.46, 95% CI = 1.33-1.62) **Figure 2** demonstrates the odds ratios for those who were exposed to Mobile *Kunji* compared to those who were unexposed by health indicators across the continuum of care. These results were similar for those who had been exposed to the audio (Dr. Anita) component alone, as well as those who had been exposed to both audio and the cards (Figure S2a in the **Online Supplementary Document**). Sensitivity analysis assessing those who were exposed compared to those who were unexposed to Mobile *Kunji*, regardless of whether they were in the eight focus districts or the 30 non-focus/scale-up districts, showed similar results. The ORs between rounds 2-5 and rounds 6-9 were similar to each other, and also to those between all rounds 2-9 (Figure S2b in the **Online Supplementary Document**).

### Usage & Engagement Study (U&E)

The U&E Study investigated the specific health-related recommendations recalled by women in the eight focus districts who were exposed to Mobile *Kunji* compared to those who were unexposed. Messages conveyed to currently pregnant women differed from those who had recently given birth, and thus the messages recalled by each cohort of women were compared separately. Pregnant women who were exposed reported significantly higher message recall across the primary topics surveyed, with the exception of emergency preparedness (**Table 4**). Among women who recently gave birth, messages on complementary feeding, family planning and handwashing were recalled more frequently in the exposed group compared to the unexposed group (**Table 4**).

**Table 4:** Messages recalled from the last two interactions with frontline workers in eight focus districts amongst currently pregnant women and recently delivered mothers who were exposed vs unexposed to Mobile *Kunji*, Usage & Engagement Study, October-December, 2014, Bihar, India

	Pregnant mothers			Recently delivered mothers		
	**Exposed to Mobile *Kunji* (n = 1193)**	**Unexposed to Mobile *Kunji* (n = 556)**	*P*-value*	**Exposed to Mobile *Kunji *(n = 1204)**	**Unexposed to Mobile *Kunji* (n = 398)**	*P*-value*
**Message recalled (%):**						
Birth preparedness	82.2	77.7	0.025	8.2	11.1	0.086
Institutional deliveries	28.7	18.0	<0.001	2.1	1.5	0.475
Newborn care	9.3	6.1	0.024	3.4	2.0	0.161
Emergency preparedness	1.9	0.9	0.11	1.8	0.3	0.022
Family planning	37.2	20.9	<0.001	58.1	60.1	0.502
Exclusive breastfeeding	3.6	1.6	0.023	13.0	8.0	0.008
Complementary feeding	26.4	18.4	<0.001	83.9	49.5	<0.001
Handwashing	21.9	5.8	<0.001	47.1	20.1	<0.001

*P-value calculated using χ^2^ test.

#### Impact of Mobile *Kunji*

Among currently pregnant women, significantly higher proportions of exposed women reported knowledge of appropriate birth preparedness practices with the exception of saving money (**Table 5**). Exposed women also more frequently registered their pregnancies (80.4% vs 75.3%, *P* < 0.01) and exclusively breastfed their infants until age 6 months (58% vs 42.5%, *P* < 0.01). Regarding family planning practices, only use of condoms was significantly higher among exposed women, although this practice was reported by only 6.4% of exposed women.

**Table 5:** Health-related knowledge and behaviours among pregnant women exposed vs unexposed to Mobile *Kunji* in eight focus districts, Usage & Engagement study, October-December, 2014, Bihar, India

	Exposed to Mobile *Kunji* (%)	Unexposed to Mobile *Kunji* (%)	*P*-value*
**Birth preparedness:**			
Sample size (n) †	1219	558	
Pregnancy registered	80.4	75.3	0.01
**Knowledge of critical things one should plan for delivery:**			
Saving money	84.2	83.7	0.8
Keep important phone numbers of health worker	43.3	26.3	<0.001
Arrange for transportation for delivery	33.2	23.3	<0.001
Arrange for transportation for emergency situations	13.5	8.8	<0.01
Identify place of delivery	32.9	41.8	<0.001
**Breastfeeding & complementary feeding:**			
Sample size (n)	1230	400	
Child exclusively breastfed until 6 months	58.0	42.5	<0.01
Knowledge that complementary feeding should be initiated at 6 months	55.9	52.0	0.17
**Family planning:**			
Sample size (n)	2423	956	
Methods of family planning ever used			
Intrauterine device	1.0	0.9	0.81
Condoms	6.4	4.0	<0.01
Oral contraceptive pill	3.7	3.7	0.99
Injectable	0.9	0.8	0.84
Female sterilisation	5.3	4.6	0.39
Male sterilisation	0.3	0.2	0.84

*P-value calculated using χ^2^ test.

†Comprised of respondents who endorsed exposure to messages addressing the topic of interest.

The U&E study additionally surveyed maternal respondents on a variety of questions related to their trust in FLWs (Table S3 in the **Online Supplementary Document**). Trust was generally higher among those exposed to Mobile *Kunji*. For example, those who were exposed to Mobile *Kunji* were significantly more likely to describe their FLWs as “completely trustworthy” on issues related to pregnancy and newborn care (93.5% vs 79%, *P* < 0.001). Similarly, exposed women were significantly more likely to “completely agree” with the information given by their FLW compared to those who were unexposed (94.4% vs 86.5%, *P* < 0.001). The duration of interaction with a FLW was almost 10 minutes longer per visit for the women who were exposed to Mobile *Kunji *(mean 21 minutes vs 13 minutes, *P* < 0.001), and a significantly higher percentage of women reported discussing the information they received with someone else, often including other family members (54.4% vs 37%, *P* < 0.001).

## DISCUSSION

Across all data sets, our evaluation showed that exposure to the Mobile *Kunji* mHealth tool implemented by BBC Media Action was associated with significantly improved knowledge and health-related behaviours for indicators across the RMNCHN continuum of care. This was particularly notable for birth preparedness and appropriate ANC practices, as well as postnatal behaviours such as exclusive breastfeeding and complementary feeding. Further, Mobile *Kunji* was shown to have significant impact on the self-efficacy of FLWs who used it, as well as on the trust of their beneficiaries in them. Results were more mixed for behaviours related to family planning and immunisations. It should be noted, however, that active issues in the federally managed supply chain for contraceptives and vaccines were reported during this time and may have mitigated impact on these practices due to difficulties in accessing them.

There were multiple limitations of this study. All three evaluations were survey-based and relied upon self-reported exposure to the interventions. The methods for the Mathematica survey were the most rigorous among the evaluations conducted, as these data were collected by an independent team under strict quality control. However, the level of exposure and impact among those who were exposed to Mobile *Kunji* may have been limited by the relatively short, two-year time period for implementation spanned by this survey. While a subsequent survey would have allowed for more time to assess the program’s impact, this was never done. The CHS data set was unique in that it was collected periodically throughout the six-year implementation period (2012-2017); however, it was originally intended to be used for internal monitoring information rather than evaluative data. The U&E surveys had limited sample sizes for those exposed to certain messages, and thus, detecting meaningful differences in the recall of some specific messages was limited. Furthermore, the U&E data was managed internally by BBC Media Action, and was dependent on maternal recall at a single time point; thus, the results may have been affected by social desirability and response biases. The differences in reported behaviours, however, were noted to be similar to the independently conducted Mathematica and CHS surveys (rounds 6-9). An important overarching limitation of this study is that the results of these surveys cannot delineate whether Mobile *Kunji* was used alongside other interventions given the context of a complex program implemented through various delivery platforms [35]. Additionally, there may have been unaccounted for clustering of health behaviours among communities and selection bias when FLWs chose those beneficiaries for whom they would utilise mHealth tools. Finally, because intensive support and facilitation were provided for FLWs by BBC Media Action during the implementation period, generalisability may be limited given the challenges for sustainability and scalability of interventions bolstered by such extensive support.

Despite these limitations, the impacts seen across multiple surveys for those women who were exposed to Mobile *Kunji* are significant and important, particularly given their implementation at scale. Many previous studies of mHealth tools have shown targeted benefits, particularly in ANC compliance and breastfeeding practices [23,31,32]. Studies of mHealth tool deployment have less often demonstrated benefits for postnatal care practices such as immunisation compliance and contraception usage [26,28,30], as shown here. Moreover, many previous studies were limited in the scale of their implementation. The mHealth tools implemented by BBC Media Action were intended to improve RMNCHN knowledge and practices across approximately 28 million people in their initial pilot [54], with subsequent scale-up statewide to more than 100 million following training of over 110 000 FLWs. Thus, the scale of implementation and the rigorous evaluation across multiple data sets provides a unique contribution to the literature on mHealth.

Access to mHealth tools in LMICs creates a rich opportunity for the delivery of quality health care through mobile interventions. To ensure mHealth tools are useful and effective, rigorous evaluations of their impacts are critically important, particularly for implementation at scale. Our analysis has shown that implementation of the mHealth tools, Mobile *Kunji* and Dr. Anita, can significantly impact exposure to health-related messaging as well as improve RMNCHN knowledge and healthy behaviours. Further study is required, however, to understand how mHealth can be utilised most effectively, and for whom. Future evaluations of the effectiveness of mHealth interventions at scale with a focus on health outcomes will be critical. Additionally, these investigations should evaluate the long-term sustainability of these benefits. Finally, close attention must be paid to the impact of technology-based interventions on health disparities. While these tools may improve access to information for even the most marginalised with phone access, disparities may widen for those without access or literacy. Ultimately, technology tools continue to create opportunities for improved health impact at scale, but their use must be evidence-based to ensure cost-effective implementation and sustained, equitable benefits.

## Additional material

Online Supplementary Document
