# Supplementary material for: Impact of mHealth interventions for reproductive, maternal, newborn and child health and nutrition at scale: BBC Media Action and the Ananya program in Bihar, India
Source: J Glob Health. 2020 Dec 19;10(2):021005. doi: 10.7189/jogh.10.021005 (PMC7758913; doi:10.7189/jogh.10.021005)
Supplement: Online Supplementary Document [file jogh-10-021005-s001.pdf]

Supplementary Tables and Figures

Supplementary Table 1a: Sample Registration System (SRS) data for India (nationwide) and Bihar (2011)

2011 Health Indicators	Bihar	India
Neonatal mortality rate	29	31
Infant mortality rate[1]	44	44
Under-5 mortality rate[1]	59	55
Maternal mortality ratio	219	167

Source: Special Bulletin on Maternal Mortality in India for MMR and for all other data: http://www.censusindia.gov.in/vital_statistics/SRS_Statistical_Report.html

Supplementary Table 1b: National Family Health Survey [NFHS-3 (2005/2006) and NFHS-4 (2015/2016)] data for India (nationwide) and Bihar

Indicator	NFHS-3 (2005-2006) Bihar	NFHS-4 (2015-2016) Bihar	NFHS-3 (2005-2006) India	NFHS-4 (2015-2016) India
Female literacy (age 15-49)	37.0%	49.6%	55.1%	68.4%
Contraception prevalence rate	34.1%	24.1%	56.3%	53.5%
4 or more antenatal care visits	11.2%	14.4%	37.0%	51.2%
Institutional delivery rate	19.9%	63.8%	38.7%	78.9%
Fully immunised children, ages 12-23 months	32.8%	61.7%	43.5%	62.0%
Children under 6 months exclusively breast fed	28.0%	53.5%	46.4%	54.9%
Iron-folic acid consumption for 100+ days	6.3%	9.7%	15.2%	30.3%

Source: National Family Health Survey, India: http://rchiips.org/nfhs/

Supplementary Table 2: Interventions implemented by BBC Media by intervention platform

Intervention Platform	Intervention Description	Target Population
**Household-level interventions**		
Mobile Kunji	Job aids for health communication and teaching	Antenatal & Postnatal Women
Dr. Anita	IVR-based audio modules paired with Mobile Kunji	Antenatal & Postnatal Women
Television advertisements	Birth spacing and birth preparedness	Antenatal & Postnatal Women
Mobile Academy	Phone-based training and education modules	Frontline Workers
Kilkari	Phone reminder service for stage specific messages	Antenatal Women
**Community-level interventions**		
Khirki Mehendiwali	Radio programme for awareness of MNCH Issues	Women & Families
Gaanth Bandh lo	Street theatre for birth preparedness	Antenatal Women
Ek Teen Do	Street theatre on family planning	Women
Pet Puja	Street theatre & tablet app for complementary feeding	Postpartum Women & Families
Gup Shup Potli	Audio education played at VHSNDs	Women
IPC Tools	Paper-based job aids to support communication	Women
**Facility-level interventions**		
Diarrhoea kits	PHC and DH deployment for diarrhoea management	Women

IVR, interactive voice response; MNCH, maternal neonatal child health; VHSND, Village Health Sanitation and Nutrition Days; IPC, interpersonal communication; PHC, Primary Health Centre; DH, District Hospital

Supplementary Figure 1: Timeline comparing intervention implementation and survey data collection periods, Bihar, India

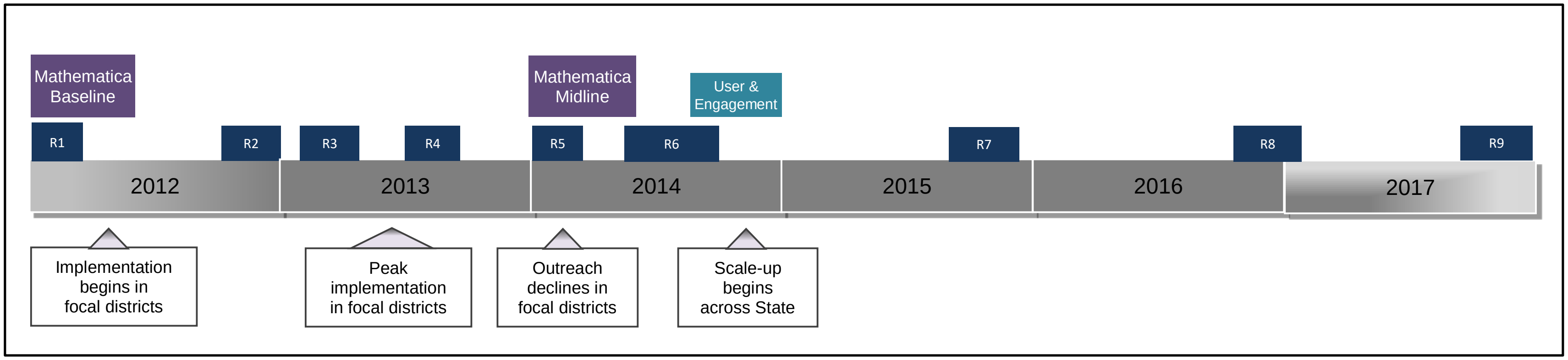

Adapted from: CARE India, *What change have we catalyzed in Bihar?* IFHI/TSU Partners' Meeting, February 2015 [unpublished]

Supplementary Figure 2a. Comparison of RMNCHN behaviours for maternal household respondents in eight focus districts who were exposed to both Mobile Kunji, Dr. Anita or both interventions compared to those who were unexposed across all rounds (2012-2017), Community-based Household Survey, Bihar, India

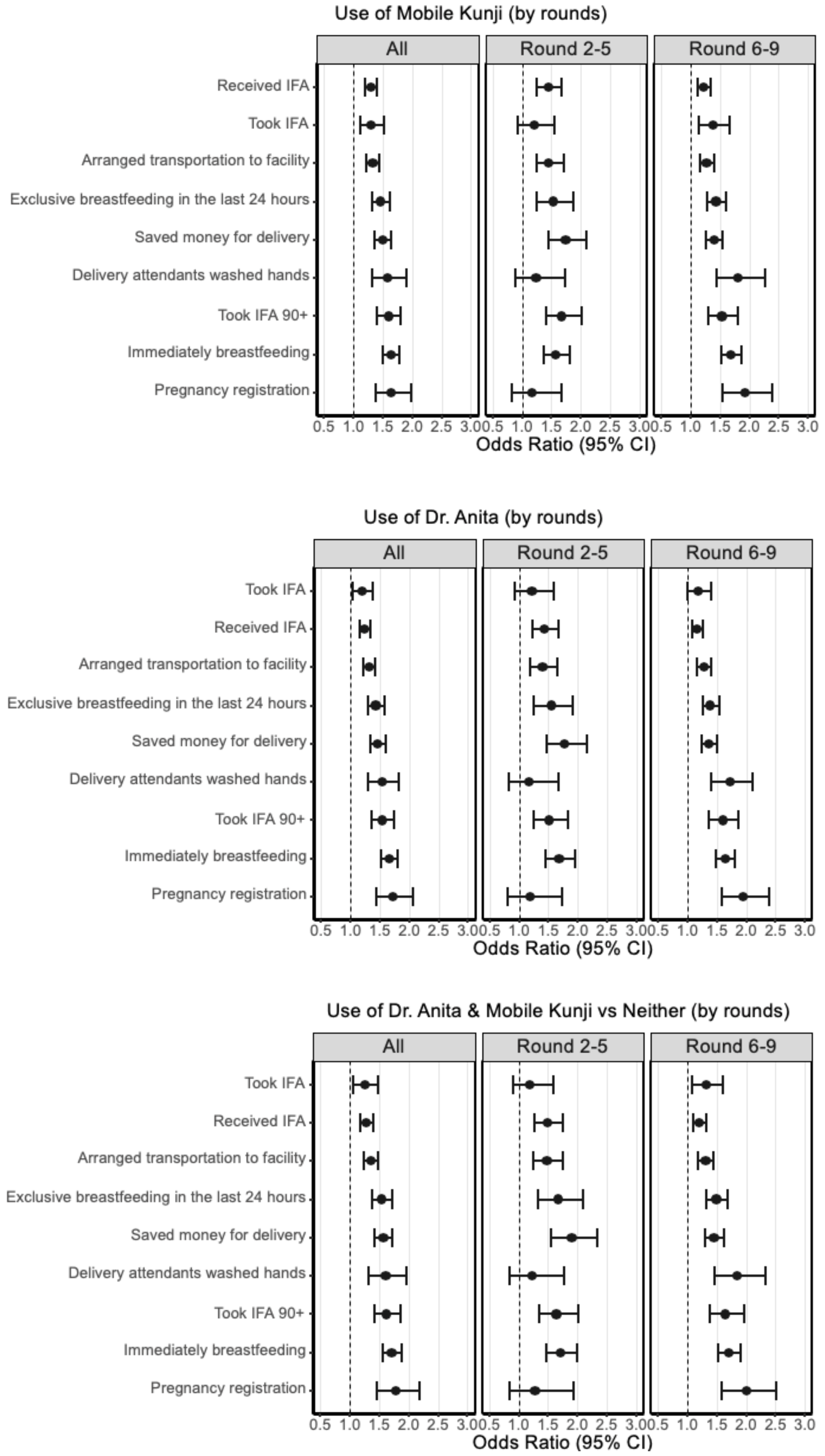

Supplementary Figure 2b: Comparison of RMNCHN behaviours for maternal household respondents in those exposed to both Mobile Kunji and Dr. Anita rounds 2-5, 6-9 and all rounds and by districts, CHS survey, Bihar, India, 2012-2017

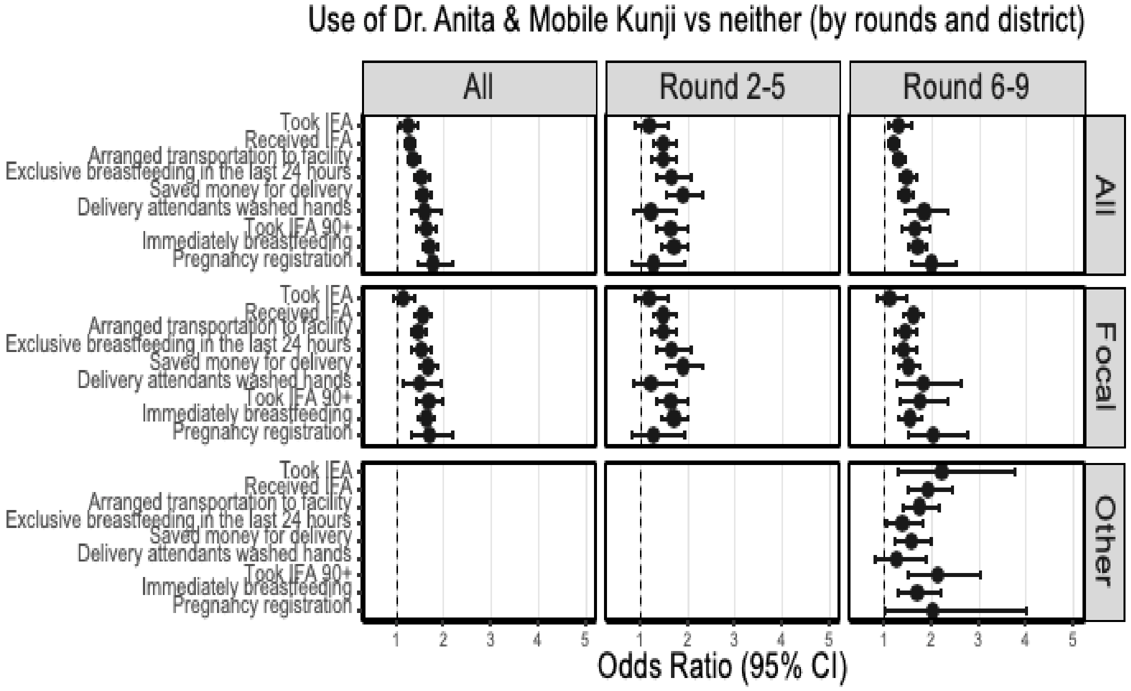

Supplementary Table 3: Impact of exposure to Mobile Kunji on household recipients' trust of frontline workers, U&E Study, Bihar, India, October-December, 2014

	Exposed to Mobile Kunji	Unexposed to Mobile Kunji	p-value*
Sample size (n)	2423	956	
Information given by the FLW differed from what the woman knew/practiced before (%)	57.5	47.4	<0.001
Extent to which woman agreed with the information given by the FLW (%)			
Completely agreed	94.4	86.5	<0.001
Partially agreed	5.5	12.6	
Did not agree	0.1	0.9	
Agreement by the woman that information received from the FLW is factually correct (%)			
Absolutely correct	94.4	81.2	<0.001
Somewhat correct	5.4	17.9	
Absolutely wrong	0.2	0.8	
Trust the FLW on issues related to pregnancy and newborn care (%)			
Completely trustworthy	93.5	78.8	<0.001
Partially trustworthy	6.2	19.3	
Not trustworthy at all	0.3	1.9	
Mean time of interaction (minutes) (mean, standard deviation)	21.1 (14.3)	12.9 (10.4)	<0.001
Discussed the information received from the FLW with someone else (%)	54.4	37.0	<0.001
Discussed information with (%)			
Husband	24.5	16.5	<0.001

Mother in law	14.9	11.3	0.006
Any family member	34.7	23.6	<0.001
Any outsider	3.1	1.5	<0.002
Trust the information provided by the FLW (%)			
Strongly Agree	64.7	43.5	<0.001
Agree	29.5	42.9	
Disagree	3.7	6.2	
Strongly Disagree	2.0	4.0	
No response	0.0	3.5	
In my community, FLWs are respected for the work they do (%)			
Strongly Agree	55.4	39.4	<0.001
Agree	39.4	42.1	
Disagree	2.3	6.6	
Strongly Disagree	2.0	4.6	
No response	0.9	7.3	
The FLW visiting my house also interacted with other family members to convince them about correct information related to pregnancy and child health (%)			
Strongly Agree	65.6	38.8	<0.001
Agree	27.3	34.3	
Disagree	5.2	11.9	
Strongly Disagree	1.5	8.8	
No response	0.4	6.2	

*P-value calculated using chi-square tests
